# Weight Loss Improves β-Cell Function in People With Severe Obesity and Impaired Fasting Glucose: A Window of Opportunity

**DOI:** 10.1210/clinem/dgz189

**Published:** 2019-11-13

**Authors:** Amy E Rothberg, William H Herman, Chunyi Wu, Heidi B IglayReger, Jeffrey F Horowitz, Charles F Burant, Andrzej T Galecki, Jeffrey B Halter

**Affiliations:** 1 Department of Internal Medicine, University of Michigan, Ann Arbor, Michigan; 2 Department of Nutritional Sciences, University of Michigan, Ann Arbor, Michigan; 3 Department of Epidemiology, University of Michigan, Ann Arbor, Michigan; 4 Department of Kinesiology, University of Michigan, Ann Arbor, Michigan; 5 Department of Biostatistics, University of Michigan, Ann Arbor, Michigan; 6 Institute of Gerontology, University of Michigan, Ann Arbor, Michigan

**Keywords:** type 2 diabetes, insulin sensitivity, insulin secretion, weight loss

## Abstract

**Background:**

In people with obesity, β-cell function may adapt to insulin resistance. We describe β-cell function in people with severe obesity and normal fasting glucose (NFG), impaired fasting glucose (IFG), and type 2 diabetes (T2DM), as assessed before, 3 to 6 months after, and 2 years after medical weight loss to describe its effects on insulin sensitivity, insulin secretion, and β-cell function.

**Methods:**

Fifty-eight participants with body mass index (BMI) ≥ 35 kg/m^2^ (14 with NFG, 24 with IFG, and 20 with T2DM) and 13 normal weight participants with NFG underwent mixed meal tolerance tests to estimate insulin sensitivity (S[I]), insulin secretion (Φ), and β-cell function assessed as model-based Φ adjusted for S(I). All 58 obese participants were restudied at 3 to 6 months and 27 were restudied at 2 years.

**Results:**

At 3 to 6 months, after a 20-kg weight loss and a decrease in BMI of 6 kg/m^2^, S(I) improved in all obese participants, Φ decreased in obese participants with NFG and IFG and tended to decrease in obese participants with T2DM, and β-cell function improved in obese participants with NFG and tended to improve in obese participants with IFG. At 2 years, β-cell function deteriorated in participants with NFG and T2DM but remained significantly better in participants with IFG compared to baseline.

**Conclusions:**

Short-term weight loss improves β-cell function in participants with NFG and IFG, but β-cell function tends to deteriorate over 2 years. In participants with IFG, weight loss improves longer-term β-cell function relative to baseline and likely relative to no intervention, suggesting that obese people with IFG are a subpopulation whose β-cell function is most likely to benefit from weight loss.

Insulin resistance is a hallmark of obesity (1, 2). Pancreatic β-cells normally adapt to the insulin resistance of obesity by increasing insulin secretion in response to glucose and other signals (2–4). In some people, such adaptation of insulin secretion can maintain normal glucose homeostasis. However, some people with obesity and impaired β-cell adaptation to insulin resistance develop impaired fasting glucose (IFG) or type 2 diabetes mellitus (T2DM) (2, 5, 6). Subjects with obesity who lose weight, either with intensive lifestyle interventions or bariatric surgery, demonstrate improvements in glucose regulation due to both lessening of insulin resistance and improved β-cell function (7–13). However, previous studies in participants with obesity have not characterized the metabolic defects across the full range of glucose homeostasis from normal fasting glucose (NFG) to IFG and T2DM, or the impact of weight loss across this spectrum. We hypothesized that β-cell function is impaired in individuals who are very obese and have IFG and T2DM and that intensive medical weight loss will improve β-cell function towards normal in both groups.

We studied normal weight participants with NFG and participants who were severely obese and had NFG, IFG, and/or T2DM, before, 3 to 6 months after, and up to 2 years after substantial medical weight loss to better understand β-cell function and the pathophysiology of glucose intolerance in people with severe obesity. Our primary measure of β-cell function was insulin secretion (Φ) adjusted for insulin sensitivity (S[I]) based on analysis of covariance (ANCOVA). This measure of β-cell function assesses the appropriateness of insulin secretion for any given degree of insulin sensitivity.

## Methods

We recruited 58 participants with severe obesity (body mass index [BMI] ≥ 35 kg/m^2^) with NFG, IFG, or T2DM who were enrolled in the University of Michigan Weight Management Program (WMP) and 13 normal weight participants (BMI < 25 kg/m^2^) with NFG. The WMP is an intensive, 2-year behavioral intervention for adults (≥18 years of age) with severe obesity, designed to promote weight loss and weight loss maintenance in order to improve the control of cardiovascular risk factors and other weight-related comorbid health conditions (14). The program is multidisciplinary and involves endocrinologists, dietitians, and exercise physiologists. During the 2-year program, there are 11 visits to a physician, 26 visits to a registered dietitian, and generally 1 visit to an exercise physiologist. Patients are seen by the physician for an initial assessment, every month for the first 3 months, and quarterly thereafter. Patients are seen by the dietitian weekly during the first month and monthly thereafter.

Weight loss is initiated with a very-low-energy diet (VLED) (800 kcal/day) in the form of total meal replacements (HMR, Inc., Boston, MA). Patients are asked to keep diaries to track the number of meal replacement shakes and soups they consume each day, deviations with other foodstuffs, hunger/satiety, and physical activity. Patients are also asked to gradually increase their physical activity (low to moderate intensity such as walking) to 40 minutes per day (either in single or divided bouts). Three to six months after the start of the VLED and after a weight loss of approximately 15% from baseline, regular foods are reintroduced and the intervention focuses on behavioral strategies to prevent weight regain, including a recommendation to engage in moderate to vigorous intensity physical activity for at least 60 to 90 minutes per day, at least 4 days per week.

All WMP participants are given an opportunity to “opt-in” to the program’s research component which was reviewed and approved by the Institutional Review Board of the University of Michigan and registered on ClinicalTrials.gov (NCT02043457). Participation is voluntary and all participants provided written informed consent.

Fifty-eight WMP patients with severe obesity participated in the study, 14 of whom had NFG at baseline (fasting plasma glucose [FPG] < 100 mg/dL), 24 had IFG (FPG 100-125 mg/dL), and 20 had non–insulin-treated T2DM (FPG ≥ 126 mg/dL or treatment with 1 or more antihyperglycemic medications, excluding insulin). All participants with obesity completed 3-hour mixed meal tolerance tests (Ensure original®, 220 calories, 32 g carbohydrate, 6 g fat, and 9 g protein) at baseline and again after 3 to 6 months of VLED and a 1 month period of weight stability. These 58 participants were a subset of the 265 people who enrolled in the WMP from December 2009 to May 2011 and consented to participate in research. There were no differences between the 58 participants included in this study and the 207 not included with respect to age, sex, race/ethnicity or baseline BMI (all *P* values ≥ 0.05). A subset of 27 of the 58 participants also completed mixed meal tolerance tests 2 years after baseline to assess longer term outcomes. Of the 27 participants studied at 2 years, 4 had NFG, 14 had IGT, and 9 had T2DM at baseline. All mixed meal tolerance tests were performed after 3 days of a prescribed weight maintenance diet that included 50% of calories from carbohydrate, 30% from fat, and 20% from protein. Participants were asked to refrain from exercise for 24 hours before the mixed meal tolerance test and to hold antihyperglycemic medications on the morning of the test. We also studied 13 normal weight participants with NFG who completed 3-hour mixed meal tolerance tests as a comparison group for the NFG participants with obesity.

Age and sex were self-reported. Weight was measured with a balance-beam scale, height was measured with a wall-mounted stadiometer, and BMI was calculated as weight/height squared (kg/m^2^). Blood samples were collected on ice and glucose was measured in the Michigan Diabetes Research Center (MDRC) Chemistry Laboratory using a Cobas Mira Chemistry Analyzer (Roche Diagnostics Corporation, Indianapolis, IN). Intra-assay variabilities for glucose were 2.0% at 84 mg/dL and 283 mg/dL. Inter-assay variabilities for glucose were 3.6% at 92 mg/dL and 2.8% at 310 mg/dL. Insulin was measured in the MDRC laboratory using a double-antibody radioimmunoassay using a ^125^I-human insulin tracer (Linco Research), a guinea pig anti-porcine insulin first antibody that is 68.5% cross-reactive to human proinsulin, and a goat anti-guinea pig gamma globulin (Antibodies Inc.)-PEG second antibody, and standardized against the Human Insulin International Reference Preparation. The limit of sensitivity for the insulin assay is 2.1 µIU/mL. Intra-assay and inter-assay variabilities are 2.7% and 3.8%, respectively at 25 µIU/mL.

Insulin sensitivity (S[I]) was calculated during the mixed meal tolerance test as proposed by Caumo et al (15). This method is based on the minimal model of glucose kinetics coupled with an equation describing the rate of appearance of glucose into the circulation after oral glucose ingestion. Caumo et al modeled glucose and insulin levels measured between −30 and 180 minutes, whereas we used levels measured only at 0, 15, 30, 60, 90, and 120 minutes. Insulin secretion was defined as phi (Φ) and calculated as the area under the curve (AUC) for insulin divided by the AUC for glucose measured between 0 and 60 minutes during the mixed meal tolerance test. S(I) and Φ were log transformed and geometric means and 95% confidence intervals (95% CI) were used to summarize the data. To test between group differences, we used nonparametric Wilcoxon tests for continuous variables and chi-square tests for categorical variables. To test follow-up versus baseline within-group differences in S(I), Φ, and Φ adjusted for S(I) where Φ and S(I) were expressed on a logarithmic scale, we used linear models with correlated errors with an unstructured covariance matrix, referred to as ANCOVA for repeated measures.

To assess differences in β-cell function between groups, we used ANCOVA testing on Φ with adjustment for S(I). First, we constructed an ANCOVA model with an interaction term: log Φ = study group + log S(I) + (log S(I) × study group), where log() is the logarithm base 10 transformation. Since the interaction term log S(I) × study group was not statistically significant, we removed it from the model. The simplified ANCOVA model took the form: log Φ = study group + log S(I). We defined *P* < 0.05 as the limit of statistical significance. Statistical analyses were performed with R3.1.2.

## Results

### Effect of 3 to 6 months of intensive weight loss

Fifty-eight participants with severe obesity were studied at baseline and after 3 to 6 months of VLED and subsequent weight stabilization (Table 1). Mean age was 49 years. Approximately one-half of participants were women and 90% were non-Hispanic white. At baseline, mean weight was 117 kg and BMI was 39 kg/m^2^ (Table 1). At baseline, values for age, sex, race, weight and BMI were not different across the NFG, IFG, and T2DM groups (Table 1). Fasting glucose, glucose AUC between 0 and 60 minutes, and fasting insulin were progressively higher in those with progressively more severe abnormalities of glucose metabolism (Table 1). Among participants with non–insulin-treated T2DM, the mean duration of known diabetes was 3 years (Table 1). Baseline data on the 13 normal weight participants are also shown in Table 1.

**Table 1.** Characteristics of Study Population at Baseline and After 3 to 6 Months of Medical Weight Loss by Glucose Tolerance Category

	Normal Weight	Obese (N = 58)							
	NFG	NFG		IFG		T2DM		Total	
	Baseline	Baseline	3-6 Mos	Baseline	3-6 Mos	Baseline	3-6 Mos	Baseline	3-6 Mos
Number	13	14	14	24	24	20	20	58	58
Age, years	45 ± 11	45 ± 9	──	51 ± 4	──	50 ± 6	──	49 ± 7	──
Sex	8F/5M	7F/7 M	──	9F/15M	──	11F/9M	──	27F/31M	──
Race/ethnicity	1B/12W	1B/13W	──	2B/22W	──	1A/2B/17W	──	1A/5B/52W	──
Duration of diabetes, years	──	──	──	──	──	3 ± 3	──	──	──
Weight, kg	65 ± 11	120 ± 12	98 ± 10**	118 ± 23	99 ± 18**	116 ± 19	96 ± 15**	117 ± 19	97 ± 15**
BMI, kg/m^2^	23 ± 2	39 ± 4	32 ± 3**	40 ± 5	33 ± 4**	40 ± 5	33 ± 4**	39 ± 4	33 ± 4**
Fasting glucose, mg/dL	94 ± 10	92 ± 6	88 ± 11	110 ± 7	100 ± 14**	123 ± 17	99 ± 9**	110 ± 16	97 ± 12*
Glucose AUC, min·mg/dL	7009 ± 797	6634 ± 661	6242 ± 840	7832 ± 911	7094 ± 862*	9094 ± 1666	7463 ± 1074**	7978 ± 1500	7015 ± 1032**
Fasting insulin, μIU/mL	10 ± 2	21 ± 6	13 ± 4**	27 ± 8	16 ± 5**	31 ± 12	16 ± 6**	27 ± 10	15 ± 5**

Values given as mean ± standard deviation.

Abbreviations: A, Asian, AUC, area under the curve between 0 and 60 minutes; B, black; BMI, body mass index; F, female; IFG, impaired fasting glucose; M, male; mos, months; NFG, normal fasting glucose; T2DM, type 2 diabetes; W, white.

*P < 0.05 post (3–6 mos) vs pre within group (Wilcoxon test for paired observations)

**P < 0.005 post (3–6 mos) vs pre within group (Wilcoxon test for paired observations)

At baseline and in comparison with normal weight participants with NFG, S(I) was significantly lower in obese participants with NFG (ratio of geometric means 0.32 [95% CI, 0.18, 0.57]; *P* = 0.0002), in obese participants with IFG (ratio of geometric means 0.23 [0.14, 0.39]; *P* < 0.0001), and in obese participants with T2DM (ratio of geometric means 0.23 [0.13, 0.39]; *P* < 0.0001) (Table 2). At baseline and compared to normal weight participants with NFG, Φ was significantly higher in obese participants with NFG (ratio of geometric means 1.88 [1.34, 2.64]; *P* = 0.0004) and obese participants with IFG (ratio of geometric means 1.91 [1.41, 2.58]; *P* < 0.0001). Compared with normal weight participants with NFG, the participants with obesity and T2DM did not differ with respect to Φ (ratio of geometric means 1.25 [0.91, 1.71]; *P* = 0.16) (Table 2). β-cell function did not differ significantly between obese participants with NFG and normal weight participants (ratio of geometric means 1.32 [0.96, 1.81]; *P* = 0.089), but tended to be lower with more severe degrees of glucose intolerance, namely obese participants with IFG (ratio of geometric means 1.21 [0.89, 1.65]; *P* = 0.22), and obese participants with T2DM (ratio of geometric means 0.79 [0.57, 1.08]; *P* = 0.13) (Table 2).

**Table 2.** Insulin Sensitivity (S[I]) and Insulin Secretion (Φ) at Baseline and After 3 to 6 Months of Medical Weight Loss in Obese Subjects by Glucose Tolerance Category

	Normal Weight	Obese					
	NFG	NFG		IFG		T2DM	
	Baseline	Baseline	3-6 Mos	Baseline	3-6 Mos	Baseline	3-6 Mos
N	13	14	14	24	24	20	20
S(I) (10^4^dL/kg·min/μIU·mL)	21.78 (14.91, 31.82)	6.90 (4.61, 10.32)	17.08* (11.15, 26.16)	5.02 (3.77, 6.68)	12.64* (9.36, 17.09)	4.93 (3.13, 7.74)	11.99* (9.03, 15.94)
Φ (μIU/mL per mg/dL)	0.41 (0.33, 0.51)	0.77 (0.60, 0.98)	0.44* (0.32, 0.61)	0.78 (0.66, 0.90)	0.48* (0.39, 0.59)	0.51 (0.39, 0.66)	0.42 (0.34, 0.53)

Abbreviations: IFG, impaired fasting glucose; mos, months; NFG, normal fasting glucose; T2DM, type 2 diabetes.

*P < 0.001 for ratio of geometric means at 3-to-6 months vs. baseline

**Figure 1.**
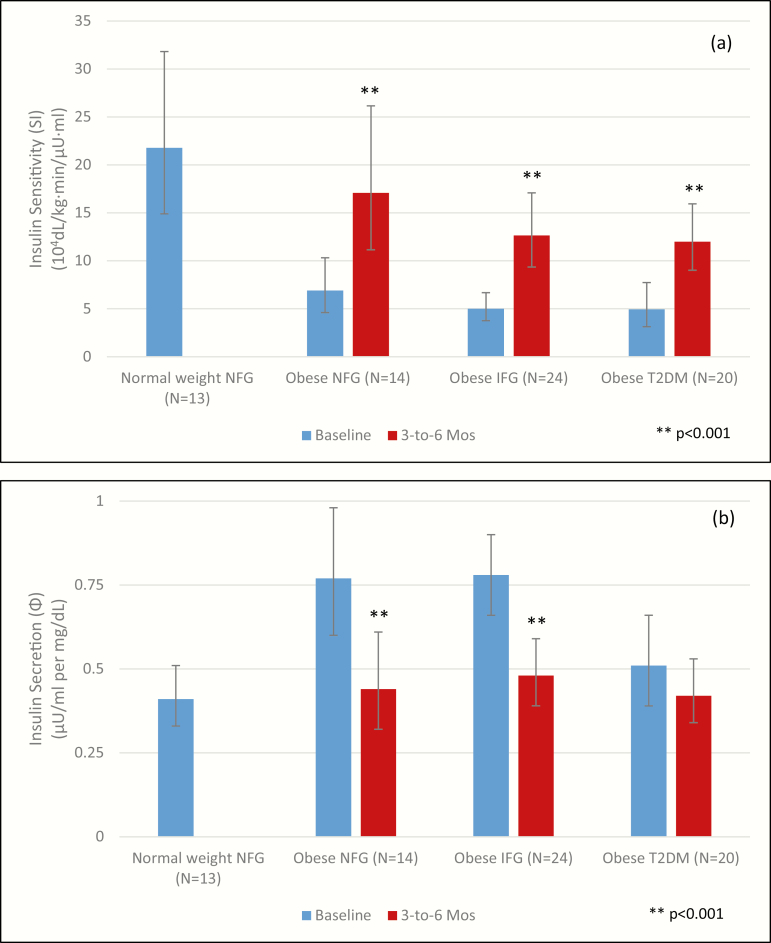
Insulin sensitivity (S[I]) (a) and insulin secretion (Φ) (b) at baseline and at 3 to 6 months by glucose tolerance category. Results are expressed as geometric means with 95% confidence intervals.

At 3 to 6 months, after a mean weight loss of approximately 20 kg and a decrease in BMI of approximately 6 kg/m^2^ (Table 1), S(I) improved significantly in obese participants with NFG (ratio of geometric means 2.48 [1.64, 3.74]; *P* = 0.0004), IFG (ratio of geometric means 2.52 [1.80, 3.53]; *P* < 0.0001) and T2DM (ratio of geometric means 2.43 [1.78, 3.33]; *P* < 0.0001) (Table 2, Fig. 1a). After weight loss, Φ decreased significantly in participants with NFG (ratio of geometric means 0.58 [0.48, 0.71]; *P* < 0.0001) and IFG (ratio of geometric means 0.62 [0.53, 0.73]; *P* < 0.0001), and tended to decrease in participants with T2DM (ratio of geometric means 0.84 [0.69, 1.01]; *P* = 0.061) (Table 2, Fig. 1b). At 3 to 6 months compared to baseline, β-cell function improved significantly in obese participants with NFG (ratio of geometric means 0.74 [0.58, 0.96]; *P* = 0.025) and tended to improve in obese participants with IFG (ratio of geometric means 0.84 [0.71, 1.00]; *P* = 0.055), but did not improve in obese participants with T2DM (ratio of geometric means 1.15 [0.91, 1.46]; *P* = 0.23).

After 3 to 6 months of weight loss, β-cell function tended to improve more in the obese NFG (ratio of geometric means 1.23 [0.94, 1.61]; *P* = 0.13) and obese IFG participants (ratio of geometric means 1.17 [0.93, 1.47]; *P* = 0.18) compared with the obese T2DM participants, but the differences were not statistically significant. At 3 to 6 months, β-cell function did not differ between obese NFG participants and normal weight participants at baseline (ratio of geometric means 0.98 [0.74, 1.31]; *P* = 0.89) or obese IFG participants and normal weight participants at baseline (ratio of geometric means 0.94 [0.72, 1.22]; *P* = 0.63) but tended to remain lower in obese T2DM participants compared to normal weight participants at baseline (ratio of geometric means 0.81 [0.61, 1.06]; *P* = 0.12).

### Effects at the 2-year follow-up assessment

Twenty-seven of the 58 participants with severe obesity who were studied at baseline and at 3 to 6 months were studied again 2 years after program initiation. The characteristics of these participants including weight, BMI, fasting glucose, and fasting insulin are shown in Table 3. The subset of participants who were studied at 2 years did not differ from those who were not studied at 2 years with respect to baseline age, sex, race, weight, BMI, fasting glucose, fasting insulin, S(I), Φ, or β-cell function (all *P* values > 0.05, data not shown).

**Table 3.** Characteristics of Study Population at Baseline, After 3 to 6 Months of Medical Weight Loss, and at 2 Years by Glucose Tolerance Category.

	Normal Weight	Obese (N = 27)											
	NFG	NFG			IFG			T2DM			Total		
	Baseline	Baseline	3–6 Mos	2 Yrs	Baseline	3–6 Mos	2 Yrs	Baseline	3–6 Mos	2 Yrs	Baseline	3–6 Mos	2 Yrs
Number	13	4	4	4	14	14	14	9	9	9	27	27	27
Age, yrs	45 ± 11	51 ± 8	──	──	50 ± 4	──	──	53 ± 6	──	──	51 ± 5	──	──
Sex	8F/5M	2F/2M	──	──	6F/8M	──	──	5F/4M	──	──	13F/14M	──	──
Race/ethnicity	1B/12W	4W	──	──	2B/12W	──	──	2B/7W	──	──	4B/23W	──	──
Duration of diabetes, yrs	──	──	──	──	──	──	──	2 ± 2	──	──	──	──	──
Weight, kg	65 ± 11	122 ± 9	99 ± 10*	108 ± 10	115 ± 24	95 ± 18*	97 ± 19*	116 ± 22	95 ± 18*	101 ± 18	116 ± 20	96 ± 16**	99 ± 17**
BMI, kg/m^2^	23 ± 2	39 ± 3	32 ± 2*	35 ± 3	39 ± 4	32 ± 3**	33 ± 4**	41 ± 6	33 ± 6*	35 ± 4*	39 ± 4	32 ± 4**	34 ± 4**
Fasting glucose, mg/dL	94 ± 10	97 ± 1	99 ± 12	102 ± 16	111 ± 6	98 ± 17**	103 ± 10*	121 ± 23	100 ± 8**	105 ± 28	112 ± 16	99 ± 13**	103 ± 18*
Glucose AUC, min·mg/dL	7009 ± 797	6771 ± 547	6923 ± 1183	7112 ± 761	7953 ± 785	7131 ± 983*	7163 ± 997	8704 ± 2041	7387 ± 1401	7971 ± 2185	8028 ± 1425	7185 ± 1129*	7425 ± 1479
Fasting insulin, μIU/mL	10 ± 2	23 ± 9	14 ± 5	13 ± 2*	28 ± 9	15 ± 6**	16 ± 8**	33 ± 15	17 ± 8*	20 ± 9*	29 ± 12	16 ± 6**	17 ± 8**

Values given as mean ± standard deviation.

Abbreviations: A, Asian, AUC, area under the curve between 0 and 60 minutes; B, black; BMI, body mass index; F, female; IFG, impaired fasting glucose; M, male; mos, months; NFG, normal fasting glucose; T2DM, type 2 diabetes; W, white; yrs, years.

*P < 0.05 post (3-6 mos or 2 years) vs pre within group (Wilcoxon test for paired observations)

**P < 0.005 post (3-6 mos or 2 years) vs pre within group (Wilcoxon test for paired observations)

**Figure 2.**
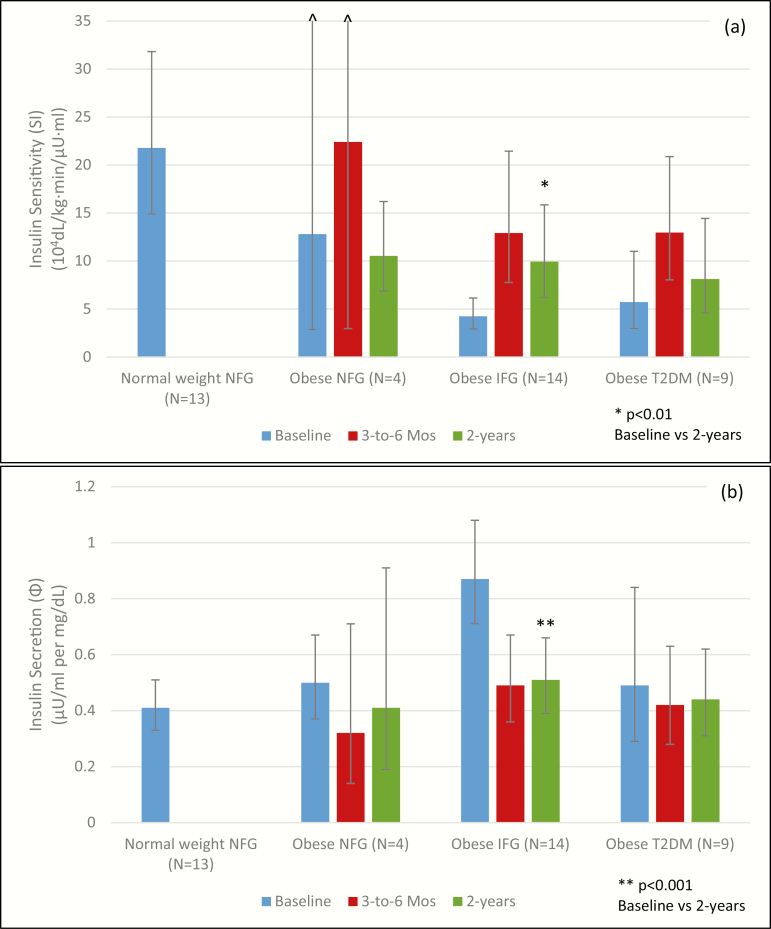
Insulin sensitivity (S[I]) (a) and insulin secretion (Φ) (b) at baseline, 3 to 6 months, and 2 years by glucose tolerance category. Results are expressed as geometric means with 95% confidence intervals.

Between 3 to 6 months and 2 years, despite maintaining a meaningful weight loss compared with baseline body weight (~10 kg weight loss), S(I) tended to decrease and Φ remained largely unchanged in the NFG, IFG, and T2DM groups (Table 4, Figs 2a and 2b). Relative to baseline, S(I) remained significantly higher (ratio of geometric means 2.34 (1.45, 3.79), *P* = 0.0021) and Φ remained significantly lower (ratio of geometric means 0.58 (0.48, 0.70), *P* < 0.0001) in participants with IFG. In obese participants with NFG and T2DM, S(I) and Φ did not differ significantly at 2 years compared with baseline. At 2 years, β-cell function remains significantly better in obese participants with IFG (ratio of geometric means 0.77 (0.64, 0.92), *P* = 0.0076) compared with baseline but was not significantly improved in obese participants with NFG (ratio of geometric means 0.82 (0.41, 1.61), *P* = 0.40) or T2DM (ratio of geometric means 0.95 (0.66, 1.35), *P* = 0.73).

**Table 4.** Insulin Sensitivity (S[I]) and Insulin Secretion (Φ) at Baseline, After 3 to 6 Months of Medical Weight Loss, and at 2 Years in Obese Subjects by Glucose Tolerance Category

	Normal Weight	Obese								
	NFG	NFG			IFG			T2DM		
	Baseline	Baseline	3-6 Mos	2 Yrs	Baseline	3-6 Mos	2 Yrs	Baseline	3-6 Mos	2 Yrs
Number	13	4	4	4	14	14	14	9	9	9
S(I), 10^–4^ dL/kg ·min/μIU·mL	21.78 (14.91, 31.82)	12.81 (2.88, 56.90)	22.40 (2.96, 169.58)	10.54 (6.85, 16.20)	4.24 (2.92, 6.15)	12.91 (7.77, 21.45)	9.93* (6.22, 15.85)	5.73 (2.98, 11.01)	12.96 (8.04, 20.88)	8.14 (4.59, 14.44)
Φ, μIU/mL per mg/dL	0.41 (0.33, 0.51)	0.50 (0.37, 0.67)	0.32 (0.14, 0.71)	0.41 (0.19, 0.91)	0.87 (0.71, 1.08)	0.49 (0.36, 0.67)	0.51** (0.39, 0.66)	0.49 (0.29, 0.84)	0.42 (0.28, 0.63)	0.44 (0.31, 0.62)

Abbreviations: IFG, impaired fasting glucose; mos, months; NFG, normal fasting glucose; T2DM, type 2 diabetes; yrs, years.

*P < 0.01 for ratio of geometric means at 2 years vs baseline

**P < 0.001 for ratio of geometric means at 2 years vs baseline

## Discussion

As expected, we found that participants with severe obesity (BMI ≥ 35 kg/m^2^) and NFG, IFG, or T2DM, had significantly lower (S(I) (Fig. 1a) but higher insulin secretion (Φ) (Fig. 1b) compared with normal weight participants who had NFG. β-cell function did not differ significantly between obese participants with NFG, IFG, and T2DM versus normal weight participants with NFG.

The 3- to 6-month intensive medical weight loss intervention substantially reduced weight and improved S(I), but generally not to the level observed in normal weight participants with NFG (Table 2, Fig. 1a). The improvements in S(I) tended to be greater in participants with obesity and NFG than among those who had IFG or T2DM (Table 2, Fig. 1a). After 3 to 6 months, Φ decreased in all 3 groups with obesity to levels no different from the normal weight NFG group (Table 2, Fig. 1b). After 3 to 6 months of medical weight loss, β-cell function improved significantly in obese participants with NFG, tended to improve in obese participants with IFG, and did not improve in obese participants with T2DM. The improvements in S(I) and β-cell function that we observed were similar to the improvements observed in a clinical trial that achieved 5% or 16% weight loss versus weight maintenance in nondiabetic participants with obesity (13). Our report extends these findings to participants with obesity and IFG and T2DM. In our study, β-cell function remained lower in the participants with obesity and IFG and T2DM compared with participants with normal weight or obesity and NFG.

Our results demonstrate that in participants with severe obesity and NFG, reduced S(I) is partially compensated by increased insulin secretion and that short-term weight loss improves S(I) and decreases insulin secretion towards normal. In contrast, in participants with severe obesity and T2DM, S(I) and Φ are significantly lower at baseline and the changes in both S(I) and Φ are less than in participants with obesity and NFG. After weight loss, β-cell function tends to remain lower, suggesting that the observed increase in insulin secretion is inadequate to restore β-cell function to normal levels in participants with severe obesity and T2DM. In participants with severe obesity and IFG, the β-cell defect is less severe and β-cell function tends to return more towards normal.

Unlike previous studies of patients with severe obesity before and after bariatric surgery (7, 16, 17), we found that the improvement in S(I) with weight loss at 3 to 6 months was influenced by baseline glucose tolerance status, but insulin secretion following weight loss did not differ by baseline glucose tolerance status. While some studies have reported no statistically significant change in insulin secretion with medical weight loss in participants with NFG and IFG (6), we found that participants with severe obesity and NFG, IFG, or T2DM all had decreases in insulin secretion and achieved the same levels of insulin secretion 3 to 6 months after weight loss. More importantly, we found that β-cell function, improved at 3 to 6 months following weight loss in participants with obesity and NFG, tended to improve in participants with obesity and IFG, but did not improve significantly in participants with obesity and recent-onset T2DM. The smaller relative decrease from baseline in insulin secretion in patients with T2DM suggests that in these individuals with severe obesity, β-cells are operating at close to their functional limit. Despite similar weight loss and resultant BMIs across the groups at 3 to 6 months and despite dramatic improvements in insulin sensitivity, β-cell function in obese participants with NFG and IFG improved to levels similar to those in normal weight controls, whereas β-cell function tended to remain lower in obese participants with T2DM compared with normal weight participants. Therefore, the improvement in β-cell function at 3 to 6 months was less robust in participants who had progressively more severe abnormalities of glucose tolerance.

Interestingly, 2 years after initiation of the weight loss intervention, many of the improvements in metabolic health that we observed after 3 to 6 months of weight loss were largely reversed. The health benefits of weight loss appeared to be most persistent in patients with IFG. The persistent improvements in S(I), Φ, and β-cell function in the participants with obesity and IFG may reflect, in part, better maintenance of weight loss at 2 years. Between assessments at 3 to 6 months and the 2-year follow-up, the IFG participants regained, on average, only 2 kg of the 20 kg they had initially lost (10% weight regain) (Table 3). In contrast, the NFG group regained 9 kg of the 23 kg (39%) and the T2DM group regained 6 kg of the 21 kg (29%) they had initially lost (Table 3). The small number of NFG participants with obesity studied at 2 years (N = 4) and the variability in their weight regain also may have limited the power to see significant differences.

Taken together, our results suggest that substantial short-term weight loss can restore glucose homeostasis in individuals with severe obesity and NFG, IFG, and recent-onset T2DM not treated with insulin, with greater benefits accruing to those with less severe abnormalities of glucose metabolism. They further demonstrate that with persistent weight loss, individuals with IFG may experience longer-term benefits in glucose homeostasis. Although β-cell function declined in participants with IFG between 3 to 6 months and 2 years, it remained better than at baseline (Table 4). These results are consistent with those of the Diabetes Prevention Program that demonstrated that ~5% weight loss in participants with obesity and impaired glucose tolerance can delay or prevent progression to T2DM (18). It is less clear from our studies whether individuals with obesity and T2DM would experience persistent benefits if they maintained weight loss over 2 years.

There are a number of limitations to our study. First, we used insulin and not C-peptide to assess β-cell function. Insulin clearance may change with weight loss and changes in insulin sensitivity. Second, a relatively small number of participants were followed for the entire 2-year period. Finally, we did not gather 2-year longitudinal data to describe the natural history of β-cell function without treatment in participants with severe obesity and NFG, IFG, and recent onset T2DM. In a longitudinal study of 17 Pima Indian people with obesity whose glucose tolerance deteriorated from NFG to impaired glucose tolerance (IGT) to T2DM over 5 years, Weyer et al found that transition from NFG to IGT and from IGT to T2DM was associated with higher body weight and percent body fat and progressive decreases in β-cell function (5). In the TRIPOD study, Buchanan et al described the natural history of glucose intolerance in high-risk Hispanic women with previous gestational diabetes who were overweight and obese (19). Despite standard diet and physical activity counseling, S(I) decreased by 30%, acute insulin response to glucose decreased by 35%, and β-cell function decreased by 39% from baseline over 3 years in women randomized to the placebo group (19). Despite the fact that these studies used different methods to assess β-cell function, they both suggest that progressive β-cell dysfunction is a hallmark of untreated obesity in high-risk individuals. Thus, our findings of no decline from baseline or modest improvement in β-cell function after 2 years of intervention may underestimate the true impact of intensive medical weight loss to preserve β-cell function in people with obesity.

Our findings support the hypothesis that obesity, or the metabolic environment associated with it, causes β-cell dysfunction (20, 21). Despite their impressive weight loss with medical therapy, the participants in our study remained obese 3 to 6 months after the medical weight loss intervention and tended to regain a portion of their lost weight over 2 years. Furthermore, despite substantial weight loss maintenance, reduced S(I) and a decline of β-cell function were the norm from 3 to 6 months to 2 years of follow-up. The role of genetic predisposition to type 2 diabetes may in part explain these findings. Although improved S(I) and reduced β-cell demand appear to lead to the development of improved β-cell function at 3 to 6 months, the improvement did not appear to persist over 2 years in participants with T2DM. This suggests that there is a window of opportunity to improve β-cell function in individuals with IFG, but that there is an irreversible decline in β-cell function with time in individuals with obesity and T2DM (22).

## Abbreviations

ANCOVA: analysis of covariance
AUC: area under the curve
BMI: body mass index
FPG: fasting plasma glucose
IFG: impaired fasting glucose
NFG: normal fasting glucose
T2DM: type 2 diabetes
VLED: very-low-energy diet
WMP: Weight Management Program

## Additional Information

***Disclosure Summary:*** The authors declared no conflict of interest.

***Data availability:*** The datasets generated during and/or analyzed during the current study are not publicly available but are available from the corresponding author on reasonable request.
